# The association between three-dimensional measurement of posterior tilt angle in impacted femoral neck fractures and osteonecrosis of the femoral head

**DOI:** 10.1186/s12891-023-06874-0

**Published:** 2023-09-26

**Authors:** Bo Cong, Haiguang Zhang

**Affiliations:** Yantai Key Laboratory for Repair and Reconstruction of Bone & Joint, Yantaishan Hospital Affiliated to Binzhou Medical University, Laishan District, 10087 Keji Avenue, Yantai, 264003 China

**Keywords:** Impacted femoral neck fractures, Posterior tilt angle, Three-dimensional reconstruction, Osteonecrosis of the femoral head, Internal fixation, Risk factors

## Abstract

**Background:**

Hollow screw internal fixation is commonly used in clinical treatment of impacted femoral neck fractures. Previous studies have demonstrated a correlation between the preoperative posterior tilt angle of the femoral head and failure of internal fixation, but there are fewer studies related to the occurrence of postoperative femoral head necrosis and the posterior tilt angle.

**Methods:**

To examine the relationship between three-dimensional posterior tilt angle measurements in affected femoral neck fractures and postoperative osteonecrosis of the femoral head and related risk variables. We retrospectively examined 130 Yantaishan Hospital patients with affected femoral neck fractures from 2019 to 2022. Three-dimensional reconstruction technology assessed the posterior tilt angle of the femoral head and separated patients into necrosis and non-necrosis groups based on postoperative femoral head necrosis. Univariate analysis compared clinical data between groups, and multivariate logistic regression analyzed risk variables for femoral head necrosis. Postoperative femoral head necrosis was predicted by posterior tilt angle using Receiver operating characteristic (ROC) curve analysis.

**Results:**

Out of 130 patients who were followed up for 16–68 months postoperatively, 20 developed femoral head necrosis. Multivariate logistic regression analysis indicated that the posterior tilt angle and reduction quality level C were risk factors for the occurrence of femoral head necrosis. The ROC curve analysis showed that the three-dimensional measurement of the posterior tilt angle had predictive value for postoperative femoral head necrosis, with a cut-off value of 20.6°.

**Conclusions:**

These results suggest that, for patients with impacted femoral neck fractures, the posterior tilt angle and reduction quality level C are risk factors for femoral head necrosis following closed reduction and internal fixation surgery. Fracture patients with a posterior tilt angle ≥ 20.6°are more likely to experience postoperative femoral head necrosis.

## Introduction

The problem of population aging is particularly acute in numerous regions globally, and hip fractures have become increasingly common among the elderly. The medical community identifies these conditions as both prevalent diseases and multiple incidence diseases among the older generation, due to the high incidence, high hospitalization rate, disability, and mortality rates [1]. Over 1.7 million cases of hip fractures are reported annually, accounting for 12% of total adult fractures. Among these, femoral neck fractures account for 54% [2, 3].

Femoral neck fractures are often described clinically according to Garden's classification, which determines the severity of the displacement. Impacted femoral neck fractures fall into Garden's Type I category. These fractures often result from excessive external rotation, leading to femoral head externalization and posterior tilt, contributing to 11% to 25% of all femoral neck fractures [4, 5]. Predominantly observed in the elderly population, these injuries often occur due to accidents or violent trauma like falls or car accidents, with a significantly lower incidence rate in younger people. Although impacted femoral neck fractures are stable fractures, research has shown that conservative treatment can lead to a higher reoperation rate and secondary displacement [6]. The standard clinical treatment strategy for impacted femoral neck fractures is internal fixation with three cannulated screws. However, femoral head necrosis, the most severe postoperative complication, occurs in 10%-30% of cases [7]. This condition often causes significant pain and burden to patients. Its early manifestation is elusive and challenging to identify, and when discovered in late stages, it is often accompanied by hip arthritis, requiring joint replacement [8]. Thus, the ability to predict femoral head necrosis is of vital clinical significance.

Presently, much of the research focuses on the correlation between the posterior tilt angle after impacted femoral neck fractures and the failure of postoperative internal fixation [9–12]. ALHO et al. [10] were the first to explore this correlation in 1992. PALM et al. [12] proposed a new method for measuring the posterior tilt angle on lateral X-ray films in 2009, finding that when the tilt angle exceeded 20 degrees, the risk of reoperation for femoral neck fracture patients increased. Despite the relatively low probability of internal fixation failure, non-union, and screw cut-out in patients with impacted fractures, the occurrence rate of femoral head necrosis is relatively higher. SHIN et al. [13] used lateral X-ray films to measure the posterior tilt angle of the femoral head and found a correlation between the preoperative tilt angle and femoral head necrosis. However, their study used two-dimensional X-ray measurements, which may be limited by non-standard patient positioning and poor imaging quality.

With the advancement of digital orthopedic technology, three-dimensional reconstruction measurement techniques can enhance the precision and reliability of skeletal anatomy studies. Consequently, the innovative aspect of our study is the utilization of three-dimensional reconstruction technology to measure the posterior tilt angle of the femoral head. This research aims to explore the correlation between the posterior tilt angle of impacted femoral neck fractures and femoral head necrosis, evaluate its predictive value, and analyze the relationship between other factors and the occurrence of femoral head necrosis.

## Materials and methods

### Study design

This retrospective study was conducted from May 2019 to May 2022 at Yantaishan Hospital in Yantai, China. General information (such as age, gender, body mass index, injury side, injury mechanism, time from injury to surgery, American Society of Anesthesiologists (ASA) classification, hospital stay, occurrence of femoral head necrosis) was collected during outpatient follow-up of patients with impacted femoral neck fractures. Radiological data were reviewed, and the quality of reduction was evaluated using the Garden alignment index measured on postoperative anterior–posterior and lateral X-rays, classified into grades A, B, and C (Table 1) [14]. Patients were divided into necrosis and non-necrosis groups based on whether ischemic necrosis occurred after femoral neck fracture surgery. Clinical data from two groups were compared using univariate analysis. Multivariate logistic regression was utilized to analyze risk factors for femoral head necrosis. The predictive value of the posterior tilt angle on postoperative femoral head necrosis was assessed using the receiver operating characteristic (ROC) curve. All participants provided informed consent and the study protocol was approved by the Ethics Committee of Yantaishan Hospital (2021–014).

**Table 1:** Quality classification of femoral neck fracture reduction

Reduction Quality	Deviation Angle (°)	Neck-Shaft Angle (°)
A	< 15	125–140
B	15–30	120–125 or 140–150
C	> 30	< 120 or > 150

### Inclusion criteria

1) Patients aged over 18 years. 2) Diagnosis of a cannulated femoral neck fracture via X-ray and having undergone closed reduction and internal fixation. 3) Complete radiographic data including preoperative X-ray, spiral CT of the proximal femur, and postoperative X-ray. 4) Follow-up period exceeding 18 months.

### Exclusion criteria

1) Pathological fractures. 2) Bilateral femoral neck fractures. 3) Old fractures, defined as fractures which had started the healing process, typically beyond a few weeks post-injury. 4) Postoperative infection within 3 months. 5) Steroid-induced femoral head necrosis. 6) Alcohol-induced femoral head necrosis. 7) Patients with Developmental Dysplasia of the Hip (DDH).

### Surgical procedure

Patients were placed in supine position under general anesthesia on a traction table. After achieving satisfactory fracture reduction under continuous traction and seeing satisfactory alignment of the fracture ends on the C-arm fluoroscope, the skin in the surgical area was disinfected thrice with povidone-iodine, and surgical drapes were applied. A 3 cm lateral incision was made at the hip, and the skin, subcutaneous tissue, and fascia were incised. Three hollow screw guidewires were inserted into the femoral head at 35 cm below the greater trochanter in an inverted "T" distribution. After confirming the position and depth of the fracture and screws with the C-arm, three 7.3 mm diameter hollow screws of appropriate length were screwed in along the guidewires. The wound was flushed with saline, and after confirming there were no missing instruments or gauze, the fascia, subcutaneous tissue, and skin were sutured in layers.

### Three-dimensional reconstruction and measurement of posterior tilt angle

Preoperative CT scans of the patients in DICOM format were imported into the Mimics 17.0 software (Materialise, Belgium). Using the software's image positioning, threshold segmentation, dynamic region growth functions, and three-dimensional calculation module, proximal femur and pelvic models were generated for both sides. The software's mirroring function was used to create a mirror image model of the healthy side using two points at the pubic symphysis and the bottom of the coccyx.

After registering the mirrored model with the fractured model, the femoral head was separated, and multiple line extraction was performed, filling into a solid sphere. The sphere's center is approximately the femoral head's center, and its coordinates were recorded. Using the 3-matic software, the mirrored healthy proximal femur model was standardized in the axial, sagittal, and coronal positions. The femoral neck's upper and lower plane boundaries were determined, and the femoral head and shaft were separated to define the femoral neck area. In defining the femoral neck area, we considered the region bounded by the superior and inferior cortical boundaries of the femoral neck. The femoral neck's center was established by creating the centroid "point." This centroid point is calculated by taking the geometric center of the defined femoral neck area, which is a standard procedure in orthopedic biomechanics to represent the center of an irregularly shaped object. It is derived from the spatial distribution of the segmented femoral neck area, and it minimizes the sum of squared distances to all other points in the region. This method provides an objective, reproducible, and reliable way of determining the center of the femoral neck. Two femoral head centers were created based on the previously recorded femoral head center coordinates. The selection of two femoral head centers accounts for potential discrepancies between the actual center and the approximated center of the femoral head as determined by the sphere. This is an additional step to enhance the accuracy of our measurements. The angle between the two femoral head centers and the femoral neck center was measured as the posterior tilt angle.

### Imaging diagnosis of osteonecrosis of the femoral head

Outpatient follow-up, combined with the patient's X-ray and MRI, was used for diagnosis. Early X-ray signs include femoral head sclerosis, cystic changes, and subchondral bone "crescent sign," whereas late signs include femoral head collapse and narrowed joint space. On MRI, T1WI linear low signal or T2W2 "double-line sign" was suggestive of osteonecrosis.

### Statistical analysis

Statistical analysis was performed using IBM SPSS 26.0. Chi-square test was used to compare factors such as gender, age, body mass index, injury side, and injury mechanism between the necrosis and non-necrosis groups. An independent samples t-test was used to compare whether there was a significant difference in the posterior tilt angle between the necrosis and non-necrosis groups. Risk factors with *p* < 0.1 in univariate regression analysis were included in the multivariate logistic regression analysis to evaluate the risk factors for ischemic necrosis of the femoral head, represented by odds ratios (OR) and 95% confidence intervals (CI). The predictive role of the posterior tilt angle for femoral head necrosis was evaluated by receiver operating characteristic (ROC) curve and calculating the area under the curve and cutoff values. A *P*-value < 0.05 was considered statistically significant.

## Results

### Participant analysis

**Fig. 1:**
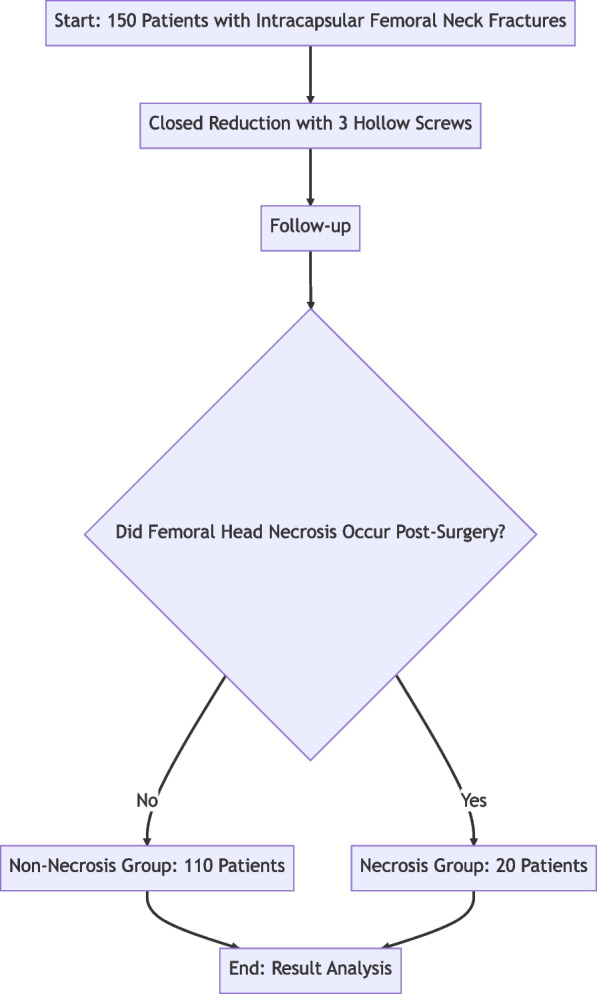
Study flowchart

Our study encompassed a cohort of 130 patients, all of whom had sustained intracapsular femoral neck fractures. These patients were followed up for a period ranging from 12 to 48 months, providing a comprehensive longitudinal view of their postoperative progress. The 130 patients in our study had hospital stays varying from 5 to 22 days, with an average duration of 8.6 days. By May 2023, 20 patients (13.3% of the total) had developed ischemic necrosis of the femoral head following internal fixation surgery. This group comprised 4 males and 16 females, aged between 47 and 72 years (average 54.4 years). When comparing the necrosis and non-necrosis groups, we found no significant differences in terms of gender, age, body mass index, side of injury, injury mechanism, time from injury to surgery, and length of hospital stay (*P* > 0.05). However, significant differences were observed in the ASA classification and quality of fracture reduction (*P* < 0.05). These findings are detailed in Table 2. The flow of the trial, including the stages of patient recruitment, intervention, and follow-up, is depicted in Fig. 1.

**Table 2:** Single factor analysis of femoral head necrosis after impacted femoral neck fracture

Indicator	Osteonecrosis Group (*n* = 20)	Non-Osteonecrosis Group (*n* = 110)	χ2 or t Value	*P* Value
Gender (n)			3.32	0.069
Male	4	45		
Female	16	68		
Age (n)			0.008	0.928
≥ 60 years old	9	49		
< 60 years old	11	61		
Body Mass Index (n)			0.58	0.449
≥ 25 kg/m2	8	27		
< 25 kg/m2	12	83		
Injury Side (n)			0.051	0.819
Left	10	60		
Right	10	54		
Injury Mechanism (n)			0.355	0.551
High-energy Injury	5	7		
Low-energy Injury	15	103		
Time from Injury to Surgery (n)			0.27	0.604
≥ 7 d	2	14		
< 7 d	18	96		
ASA Classification (n)			8.40	0.003
I, II Level	8	88		
III, IV Level	12	24		
Hospital Stay (n)			0.45	0.502
≥ 14 d	13	80		
< 14 d	7	30		
Reduction Quality (n)			6.01	0.044
A	8	86		
B	6	15		
C	6	9		
Posterior Tilt Angle (x- ± s, °)	22.93 ± 8.61	15.45 ± 7.36	4.21	< 0.001

### Three-dimensional reconstruction measurement of femoral head posterior tilt angle

**Fig. 2:**
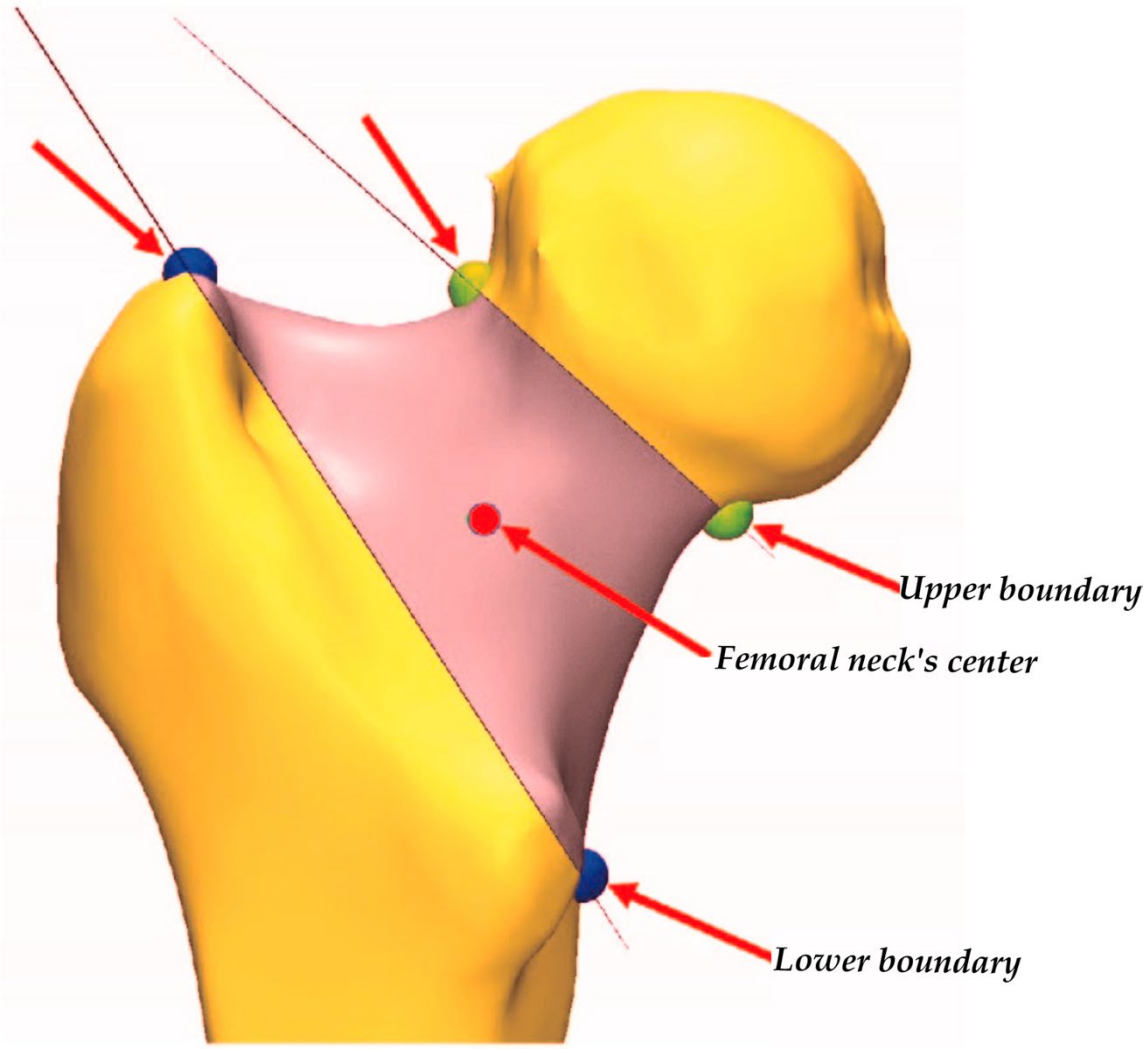
Representation of the creation of the upper and lower boundaries of the femoral neck, with the arrows indicating the selected points defining these boundaries. The central point of the femoral neck is consequently established

**Fig. 3:**
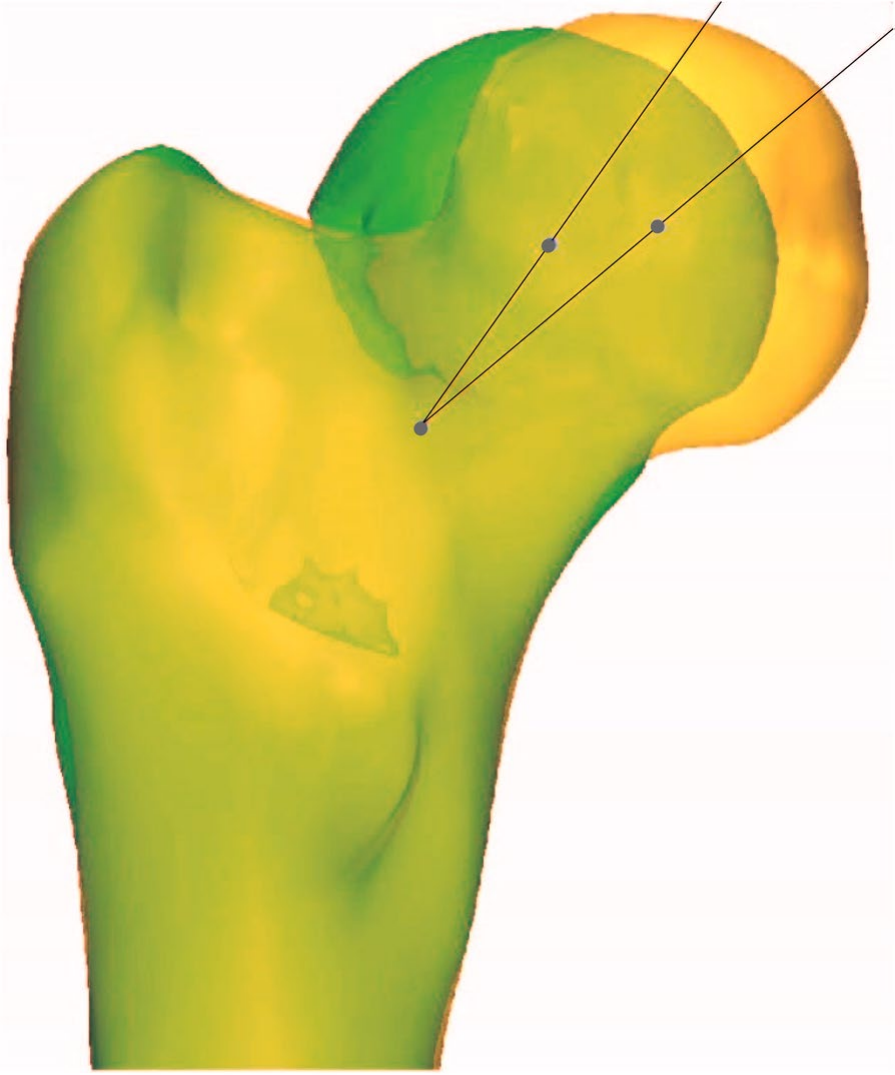
Illustration of the measurement of the posterior inclination angle of the femoral head

Using three-dimensional reconstruction, we measured the posterior tilt angle of the femoral neck fracture in patients, which ranged from 1.35° to 47.36°, with an average of (16.36 ± 7.86) °. The average posterior tilt angle in the necrosis group was (22.93 ± 8.61) °, significantly higher than that in the non-necrosis group (15.45 ± 7.36) °. This difference was statistically significant (t = 4.21, *P* < 0.001) (Figs. 2 and 3). These results are presented in Table 2.

### Results of multivariate logistic regression analysis

We included variables with a *P* value < 0.1 in the univariate regression analysis (ASA classification, quality of fracture reduction, posterior tilt angle) in the multivariate logistic regression analysis. The results indicated that the posterior tilt angle [OR = 1.125, 95%CI (1.040, 1.216), *P* < 0.05]f and C-level fracture reduction quality [OR = 5.605, 95%CI (1.510, 20.820), *P* < 0.05] were significantly associated with the development of femoral head necrosis. These findings are detailed in Table 3.

**Table 3:** Multivariate logistic regression analysis of influencing factors of femoral head necrosis

Influencing Factors	β	Wald Value	*P* Value	OR Value	95%CI
ASA Classification	0.792	1.916	0.166	2.207	0.748, 6.674
Reduction Quality B vs. A	0.639	0.895	0.344	1.894	0.530, 7.024
Reduction Quality C vs. A	1.756	6.592	0.010	5.605	1.510, 20.820
Posterior Tilt Angle	0.118	9.094	0.003	1.125	1.040, 1.216

### ROC curve analysis of femoral head posterior tilt angle predicting femoral head necrosis

**Fig. 4:**
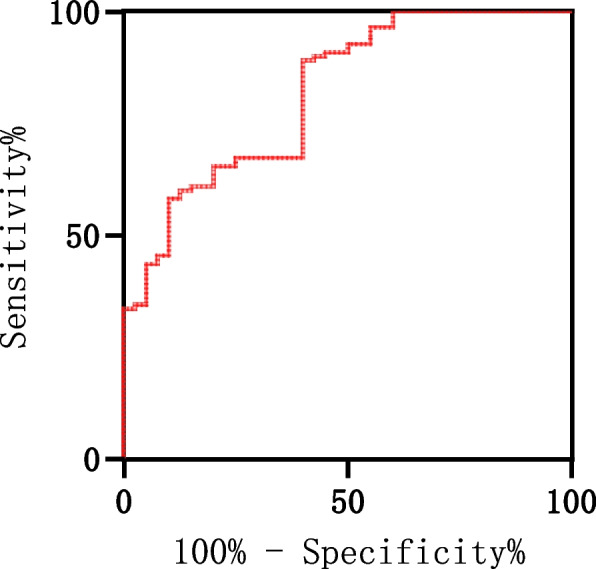
Receiver operating characteristic curve of posterior tilt of femoral head in predicting femoral head necrosis

The ROC curve, shown in Fig. 4, indicates that the posterior tilt angle measured by three-dimensional reconstruction is a significant predictor of femoral head necrosis after internal fixation surgery. The area under the curve was 0.769 [95%CI: (0.654, 0.865)]. The optimal cut-off point was 20.6°, with a sensitivity of 71% and a specificity of 79.6%. This suggests that when the femoral head posterior tilt angle exceeds 20.6°, the risk of femoral head necrosis significantly increases.

## Discussion

With advancements in internal fixation techniques and surgical proficiency, the treatment of femoral neck fractures has significantly improved. The occurrence rate of nonunion has markedly decreased, yet the incidence of femoral head necrosis remains high. Thus, early anatomical reduction, hollow nail firm fixation, and early rehabilitation are the guiding principles in treating femoral neck fractures. This study aimed to explore the occurrence of femoral head necrosis through radiological angle measurement, adopting a three-dimensional reconstruction technique for a more accurate and reliable posterior tilt angle measurement. Notably, it was found that the posterior tilt angle and Garden type C quality reduction were risk factors for femoral head necrosis after internal fixation surgery in patients with impacted femoral neck fractures [15, 16].

Two prevailing theories explain the mechanism of femoral head necrosis. One focuses on blood supply, suggesting that the primary blood supply vessels to the femoral head are the medial and lateral circumflex femoral arteries. Trauma can cause vascular injury and deformation, leading to hematoma formation within the joint capsule. The increased intra-capsular pressure exacerbates the ischemic state of the vessels, reducing the localized blood supply to the femoral head and leading to ischemic necrosis [17, 18]. The second theory centers on biomechanics. It posits that bone trabeculae need to adapt to the stress stimuli in the surrounding environment. The intense muscular contractions of the hip joint exert significant shearing forces on the fracture ends, increasing the instability of the fracture. Long-term shear forces can stimulate the trabeculae to realign, and if the realigned trabeculae cannot adapt to the stresses imposed by the acetabulum, it may lead to degradation, collapse, and fracture, thereby resulting in femoral head necrosis [19, 20].

This study measured the posterior tilt angle of the femoral head using preoperative CT scans and three-dimensional reconstruction technology, enabling a clear view of the femoral head's positional changes in three-dimensional space. Previous studies employed the supine horizontal projection method proposed by PALM et al. [12]to measure the posterior tilt angle on lateral X-ray films, based on the angle between the femoral head radius and the central axis of the femoral neck. This measurement method, although proven to be reliable within certain rotation and flexion ranges, could still be influenced by anatomical variations, osteoporosis, and obesity-induced poor image quality [21]. This study measured the posterior tilt angles in 130 cases of impacted femoral neck fractures, with an average of (16.40 ± 7.89) °. The necrosis group exhibited significantly larger posterior tilt angles than the non-necrosis group (*P* < 0.05). Multifactor logistic regression analysis identified the posterior tilt angle as a risk factor for femoral head necrosis post-internal fixation surgery.

The present study involved 130 cases of impacted femoral neck fractures. The occurrence rate of postoperative femoral head necrosis was 13.3%, which aligns with foreign literature reports [22, 23]. This study identified the posterior tilt angle as a risk factor for femoral head necrosis. The ROC curve analysis revealed a good predictive value of the posterior tilt angle for femoral head necrosis after internal fixation surgery, suggesting that fracture patients with a posterior tilt angle ≥ 20.74° are more prone to postoperative femoral head necrosis. Shin et al. [13] identified that the posterior tilt angle on lateral X-ray was an independent predictive factor for femoral head necrosis in patients with impacted femoral neck fractures. They established through ROC curve analysis that when the femoral head posterior tilt angle exceeds 7.3°, the incidence of femoral head necrosis increases. However, their study only included patients with low energy injuries. Our research, by contrast, includes patients with high energy injuries such as falls from heights and car accidents, hence our threshold value is lower than their findings. Our study also found that poor quality of internal fixation reduction is a contributing factor to femoral head necrosis. Min et al. [24] demonstrated that patients with poor fracture reduction have a significantly higher incidence of postoperative femoral head necrosis compared to those with satisfactory reduction. Poor fracture reduction, rotation, and internal displacement of the femoral head can increase shear forces on the fracture surface, affect the reconstruction of blood vessels around the femoral head, and consequently lead to necrosis. Additionally, when fracture reduction is poor, the femoral head and acetabulum are mismatched, leading to abnormal stress distribution on the femoral head, concentrated surface stress, and degeneration, absorption, and collapse of the internal trabecular microstructure, which may also result in femoral head necrosis [25, 26]. Therefore, anatomical reduction remains the guarantee for good fracture healing after surgery for patients with impacted femoral neck fractures. Good quality reduction can not only restore blood flow reconstruction and relieve vascular compression but also maintain a normal biomechanical state and stabilize the fracture end [27, 28].

Body mass index (BMI) is an indicator to measure the degree of obesity. Patients with a higher BMI often have more pelvic muscles and greater local muscular tension at the fracture site, leading to increased fracture stress and, consequently, an increased risk of non-union and femoral head necrosis. Additionally, patients with high BMI are more likely to develop hyperlipidemia, leading to thicker blood that is prone to forming fat emboli, blocking the reconstruction of new blood vessels, and affecting the blood supply around the femoral head. The ASA grade assesses preoperative patient condition and anesthesia risk; patients with ASA grade III or IV usually have more comorbidities and higher anesthesia risk. Pei et al. [29] found that a BMI > 25 kg/m2 and ASA grades (III and IV) were risk factors for postoperative femoral head necrosis. However, our study did not find a significant correlation between BMI and ASA grade and femoral head necrosis, which may be due to our study focusing on impacted fractures, in contrast to Pei's research that included Garden I-IV fractures. The relationship between BMI, ASA grade, and postoperative femoral head necrosis may be related to sample size, fracture classification, among other factors, and needs further investigation.

This study did not find a correlation between patient age, time from injury to surgery, and the occurrence of femoral head necrosis. Traditional views suggest that younger patients, due to higher bone mass, high energy injuries, and severe vascular injury, are at higher risk of femoral head necrosis than older patients. For younger individuals presenting with severe trauma or other risk factors, considering an immediate Total Hip Arthroplasty (THA) might be prudent when the risk of femoral head necrosis is elevated [30]. Such a strategy is underpinned by the understanding that the potential advantages of THA, including pain relief, enhanced joint functionality, and possibly evading the protracted complications tied to femoral head necrosis, might surpass the downsides of undergoing joint replacement at a younger age [31]. Nevertheless, it's crucial to juxtapose these benefits against the possible challenges linked with THA in younger demographics, such as the durability of the prosthesis and the potential need for future revision surgeries.

However, in our study, the incidence of femoral head necrosis in patients under 60 (13.1%) was not significantly different from patients over 60 (13.6%). The impact of time from injury to surgery on the incidence of femoral head necrosis remains controversial. Szita et al. [32] reported that the necrosis rate was significantly lower in patients who had surgery within 6 h after injury compared to those who had surgery after 24 h. Pei et al. [29] found the probability of femoral head necrosis in the group operated on within 24 h post-injury (16.5%) was similar to that in the group operated on after 24 h (15.8%). They argued that the blood supply to the femoral head is primarily affected by the force of the injury at the time of trauma, regardless of when surgery is performed. Although our study did not find that the time from injury to surgery influenced the probability of necrosis in impacted fractures, we still recommend early internal fixation treatment to alleviate patient pain and reduce the possibility of secondary damage to the femoral head vessels.

Our study presents several limitations that need consideration. Firstly, as a retrospective study, there might be some selection bias despite our strict quality control measures. Secondly, the sample size was limited, which can affect the generalizability of our findings. Thirdly, while our follow-up period ranged from 12 to 48 months, femoral head necrosis might manifest years after osteosynthesis. As such, our follow-up may not have captured all potential cases. This is supported by studies suggesting that femoral head necrosis can develop up to 5 years or longer post-trauma, particularly among patients who initially showed no symptoms. We also recognize the potential limitations related to the use of the posterior tilt angle as a key metric. While our study aimed to introduce and emphasize this novel metric's value, we appreciate that it might act as a proxy for more global concerns related to blood supply disruption and integrity. It's essential to interpret this metric in light of these broader physiological and pathological contexts, ensuring a comprehensive understanding of its implications in femoral head necrosis.

Furthermore, the surgical experience across different surgeons could influence postoperative complication rates. In terms of validation, our study lacks internal validation as we didn't partition our dataset for separate training and validation. This is an area future research should address to bolster the findings' robustness. Additionally, our conclusions rely solely on our dataset and haven't undergone external validation with independent data. Hence, while we believe our results provide important insights, their applicability needs verification in larger and more diverse cohorts.

## Conclusions

In conclusion, for patients with impacted femoral neck fractures, the posterior tilt angle and reduction quality level C are risk factors for femoral head necrosis following internal fixation surgery. Through three-dimensional reconstruction technology, the posterior tilt angle of the femoral head can be accurately measured. Fracture patients with a posterior tilt angle ≥ 20.6° are more likely to experience postoperative femoral head necrosis.

## Data Availability

The datasets used and/or analyzed during the present study are available from the corresponding author on reasonable request.
